# Supercoiling of an excised genomic island represses effector gene expression to prevent activation of host resistance

**DOI:** 10.1111/mmi.14111

**Published:** 2018-10-03

**Authors:** Helen C. Neale, Robert W. Jackson, Gail M. Preston, Dawn L. Arnold

**Affiliations:** ^1^ Centre for Research in Bioscience, Faculty of Health and Applied Sciences The University of the West of England, Frenchay Campus Bristol BS16 1QY UK; ^2^ School of Biological Sciences University of Reading Reading RG6 6UR UK; ^3^ Department of Plant Sciences University of Oxford Oxford OX1 3RB UK

## Abstract

The plant pathogen *Pseudomonas syringae* pv. *phaseolicola*, which causes halo blight disease of beans, contains a 106 kb genomic island PPHGI‐1. PPHGI‐1 carries a gene, *avrPphB,* which encodes an effector protein that triggers a resistance response in certain bean cultivars. Previous studies have shown that when PPHGI‐1 is excised from the bacterial chromosome, *avrPphB* is downregulated and therefore the pathogen avoids triggering the host’s defence mechanism. Here, we investigate whether the downregulation of *avrPphB* is caused by the supercoiling of PPHGI‐1. We also investigate the effect of a PPHGI‐1‐encoded type 1A topoisomerase, TopB3, on island stability and bacterial pathogenicity in the plant. Supercoiling inhibitors significantly increased the expression of *avrPphB* but did not affect the excision of PPHGI‐1. An insertional mutant of *topB3* displayed an increase in *avrPphB* expression and an increase in PPHGI‐1 excision as well as reduced population growth in resistant and susceptible cultivars of bean. These results suggest an important role for topoisomerases in the maintenance and stability of a bacterial‐encoded genomic island and demonstrate that supercoiling is involved in the downregulation of an effector gene once the island has been excised, allowing the pathogen to prevent further activation of the host defence response.

## Introduction

Genomic islands are distinct regions of DNA that are present in some strains of bacteria but not others, often containing genes that may be responsible for recombination and mobility such as integrases, and are normally associated with specific integration sites in the genome such as tRNA loci (Hacker and Kaper, 2000; Hacker and Carniel, 2001; van der Meer and Sentchilo, 2003). The plant pathogen *Pseudomonas syringae* pv. *phaseolicola* (*Pph*) strain 1302A (*Pph* 1302A), which causes halo blight disease of bean, contains a 106 kb genomic island PPHGI‐1, which encodes 100 genes. PPHGI‐1 can exist in one of two forms: (i) it can be integrated within a tRNA_LYS_ locus in the chromosome; (ii) it can excise to form a circular molecule. PPHGI‐1 also contains an oriV, which enables it to become a self‐replicating plasmid (Pitman *et al*., 2005; Neale *et al*., 2013).

PPHGI‐1 carries an effector gene *avrPphB* (also called *hopAR1*) that is specifically upregulated during infection. AvrPphB is then translocated via the type III secretion system from the bacterial cell into the plant cell where its cysteine protease activity is used to target cytoplasmic kinases to disrupt host immunity (Zhu *et al*., 2004; Zhang *et al*., 2010); however, in resistant bean plants, avrPphB is recognised by the resistance protein R3 (Jenner *et al*., 1991; Jackson *et al*., 2000; Arnold, *et al*., 2003). When this recognition occurs, the plant cells undergo a hypersensitive response (HR), also known as effector‐triggered immunity, which is a form of programmed cell death (Jones and Dangl, 2006). This leads to an antimicrobial environment at the site of infection, death of the invading bacteria and therefore the plant resists further pathogen infection and spread within the plant (Alfano and Collmer, 2004; Jones and Dangl, 2006). During the HR, it has been observed that an integrase gene (*xerC*) on PPHGI‐1 is upregulated allowing the island to excise from the chromosome and form a circular structure (Pitman *et al*., 2005; Neale *et al*., 2013). PPHGI‐1 can then be lost from the bacterial cell, leading to the loss of the effector gene. Once the effector gene is lost from the bacterial population, the bean plant no longer recognises the invading pathogen, which can rapidly replicate inside the plant and cause disease (Pitman *et al*., 2005; Lovell *et al*., 2009). However, an interesting inverse gene expression state exists between the chromosomally located PPHGI‐1 and the excised form: it was observed that when PPHGI‐1 is in its excised circular form, *avrPphB* is downregulated. This suggests that the circular form of PPHGI‐1 affects gene expression (Godfrey *et al*., 2011), possibly by supercoiling, and that the reduction in gene expression due to supercoiling may be a way of masking the presence of *avrPphB* from detection by the host surveillance system.

DNA topoisomerases are enzymes that regulate the coiling and relaxing of the DNA super helix and therefore control the topology of DNA in all cells (Bush *et al*., 2015). They are a ubiquitous family of enzymes and have important roles to play in many processes including DNA replication, chromosome segregation, recombination and repair (Luttinger, 1995, Vos *et al*., 2011, Bush *et al*., 2015). Topoisomerases can also be recruited to active transcriptional units to remove negative supercoils and influence transcription of genes (Peter *et al*., 2004; Ahmed *et al*., 2017).

Type I and type II topoisomerases introduce breaks into single‐ and double‐stranded DNA respectively (Terekhova *et al*., 2013; Bush *et al*., 2015). Bacterial type I topoisomerases can be further divided into subgroups, for example topoisomerase I and III, both of which are classed as topoisomerase type 1A enzymes (Deweese *et al*., 2008; Terekhova *et al*., 2013; Bush *et al*., 2015). Topoisomerase I removes negative DNA supercoils and works with DNA gyrase (type II topoisomerase) to regulate the level of supercoiling of the chromosomal DNA (Deweese *et al*., 2008). Topoisomerase III (topo III) enzymes are conserved and ubiquitous and resolve single‐stranded DNA recombination and replication intermediates and in some instances double‐stranded intermediates (Terekhova *et al*., 2013).

Pitman *et al*. (2005) used BLASTP analysis to show that the gene PPH.56 from *Pph* genomic island PPHGI‐1 was related to a topo III topoisomerase enzyme. PPH.56 was therefore designated *topB3*. The presence of this gene on the island may indicate the topoisomerase specifically influences island topology or expression of island‐based genes. Topoisomerase genes have been associated with other genomic islands. For example, *Pb*TopoIIIβ was described as a topoisomerase III enzyme from *Pectobacterium atrosepticum* (*Pba*) strain SCRI1043 that negatively regulates the excision of pathogenicity island HAI2 (Vanga *et al*., 2012). Inactivation of *Pb*TopoIIIβ on HAI2 caused a 10^3^‐ to 10^4^‐fold increase in excision, led to reduced fitness *in vitro* and a decrease in the virulence of *Pba* SCRI1043 in potato. It was therefore suggested that *Pb*TopoIIIβ may be required for stable maintenance of HAI2 in the chromosome of *Pba* and may control as yet unidentified genes involved in the viability and virulence of *Pba* SCRI1043 in potato (Vanga *et al*., 2012). As well as being involved in regulating genomic island topology, topoisomerase III enzymes are also associated with plasmid maintenance, for example the broad host range plasmid RP4 TraE protein exhibits topoisomerase III activity and has been found to have a role in the propagation of plasmids (Li *et al*., 1997).

We hypothesised that the excised PPHGI‐1 becomes supercoiled, leading to the downregulation of *avrPphB* and that the island‐encoded topoisomerase *topB3* has a role in this process. We investigated the effect of supercoiling inhibitors and a mutation in *topB3* on the excision of PPHGI‐1 and the expression of island‐encoded genes. The results show that the supercoiling inhibitors significantly increased the expression of *avrPphB* but did not affect the expression of the island‐encoded *xerC* integrase or the excision of PPHGI‐1. However, a mutation in *topB3* caused increased *avrPphB* expression and an increase in *xerC* expression and PPHGI‐1 excision, as well as reducing the population growth of the pathogen in its host plant. Together, these indicate an intimate relationship of topoisomerase and supercoiling in controlling island excision and gene expression.

## Results

### Effect of gyrase inhibitors on supercoiling in Pseudomonas syringae pv. phaseolicola

Supercoiling has been implicated in the downregulation of the effector gene *avrPphB* from *Pph* 1302A when PPHGI‐1 is excised from the chromosome (Godfrey *et al*., 2011). Here, supercoiling inhibitors were used to allow the investigation of relaxing of supercoiling on the expression of *avrPphB* and PPHGI‐1 function. The supercoiling inhibitors chosen were novobiocin, a member of the aminocoumarin group of antibiotics, whose mode of action is to competitively inhibit gyrase ATP binding, and ciprofloxacin, a quinolone antibiotic, which binds to both gyrase enzyme and DNA and stabilises the formation of the gyrase–DNA cleavage complex (Bush *et al*., 2015).

Firstly, the minimum inhibitory concentrations (MIC) for novobiocin and ciprofloxacin were determined for *Pph* 1302A. This was done by twofold dilution steps from 512 to 0.0625 µg/ml of each antibiotic in bacterial LB broth, inoculating each broth with 5 × 10^5^ cfu/ml of *Pph* 1302A and recording CFU/ml of *Pph* 1302A growth over 18 h (Fig. S1). Moving forward, and for convenience, values of 10 µg/ml for novobiocin and 0.5 µg/ml ciprofloxacin were selected for further work (nearest round numbers at which growth was unaffected), as at these values the growth of *Pph* 1302A was unaffected.

**Figure 1:**
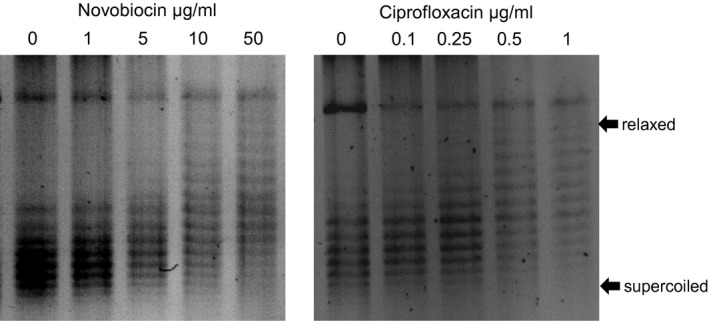
DNA relaxation by supercoiling inhibitors. Plasmid pBBR1MCS‐2, isolated from *Pseudomonas syringae* pv. *phaseolicola* 1302A, shows a dose‐dependent relaxation of supercoiling by novobiocin and ciprofloxacin. pBBR1MCS‐2 was isolated following antibiotic treatment and run on a 1% agrose gel + 2.5 µg/ml chloroquine. More negatively supercoiled topoisomers migrate faster in the gel. Direction of migration in agarose gel is from top to bottom.

Next, we tested if the selected concentrations of novobiocin and ciprofloxacin affect supercoiling in *Pph*. A supercoiling assay using chloroquine gels was performed with a *Pph* 1302A strain carrying the plasmid pBBR1MCS‐2 (Fig. 1). Topoisomers that are more negatively supercoiled migrate faster in the gel than more relaxed topoisomers (Ó Cróinín*et al*., 2006). Both novobiocin and ciprofloxacin inhibited supercoiling at the concentrations used, resulting in reduced migration of pBBR1MCS‐2.

To provide further support that the selected levels of the supercoiling inhibitors were exerting an effect on supercoiling, the expression levels of gyrase subunit B (*gyrB*) in *Pph* 1302A were measured with different treatments. GyrB is one of two subunits found in gyrase (Bush *et al*., 2015) and inhibition of GyrB by novobiocin has been shown to relax DNA supercoiling (Schröder *et al*., 2014). Novobiocin and ciprofloxacin both caused a significant (p < 0.05) increase in gene expression of *Pph* 1302A *gyrB* when measured in M9 minimal media after five hours using qPCR, whereas there was no change in the expression levels of the control gene *acpP* (Fig. S2). These results suggest that supercoiling is being affected by the inhibitors as the *gyrB* gene is being over expressed in an attempt to overcome the inhibitory effects of the antibiotics, as has also been observed in other systems (Sioud *et al*., 2009; Ferrándiz *et al*., 2010; Schröder *et al*., 2014). Overall, we can conclude that the selected concentrations of novobiocin (10 µg/ml) and ciprofloxacin (0.5 µg/ml) do not reduce cell growth but do have an effect of relaxing DNA supercoiling in *Pph* 1302A. We therefore moved on to look at the effect of relaxing supercoiling on the gene expression of PPHGI‐1‐encoded genes.

### The effect of supercoiling inhibitors on genomic island PPHGI‐1 excision and avrPphB gene expression

Godfrey *et al*. (2011) showed that the expression of the PPHGI‐1‐encoded effector gene *avrPphB* was downregulated when the genomic island was excised from the chromosome. One reason to explain this downregulation of *avrPphB* when it is on an excised circular molecule could be due to DNA supercoiling. DNA supercoiling is known to affect gene expression; for example, Ferrándiz *et al*. (2010) found that > 13% of the genome of *Streptococcus pneumoniae* exhibited relaxation‐dependent transcription. To test the hypothesis that supercoiling affected *avrPphB* expression, the effect of novobiocin and ciprofloxacin on PPHGI‐1 excision and gene expression was investigated.

**Figure 2:**
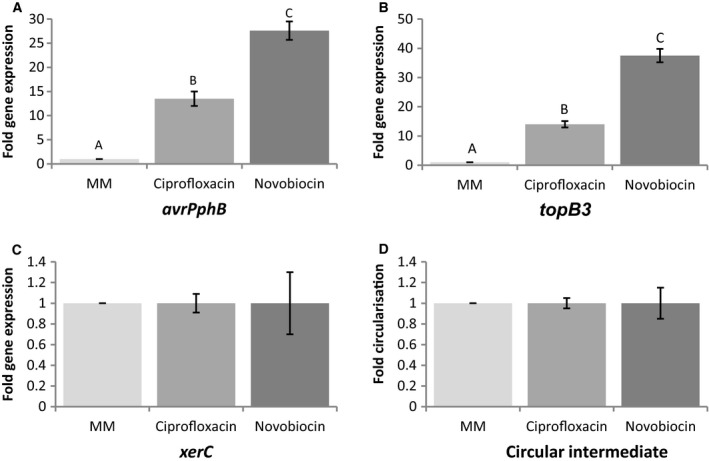
Gene expression and circular intermediate detection in *P. syringae* pv. *phaseolicola* strain 1302A after treatment with supercoiling inhibitors. *Pph* 1302A cells were examined for the effect of supercoiling (SC) inhibitors ciprofloxacin (0.5 µg/ml) and novobiocin (10 µg/ml) on the expression of A. effector gene *avrPphB*, B. topoisomerase gene *topB3*, C. integrase gene *xerC* and D. detection of the circular intermediate. Bacterial cells were incubated in M9 minimal medium (MM) plus SC inhibitors for 5 h before being treated with RNA protect and the RNA extracted (*avrPphB*,* topB3* and *xerC*) or the cells pelleted and DNA extracted (circular intermediate). Results are displayed as fold expression. *avrPphB*,* xerC* and *topB3* expressions were standardised by simultaneous qPCR analysis of *acpP* expression and error bars represent standard error of the mean of three experimental replicates. Letters above bars indicate significant differences at *p* < 0.05 assessed with Student’s *t*‐test.

*Pph* 1302A cells containing chromosomal PPHGI‐1 were treated with the supercoiling inhibitors ciprofloxacin and novobiocin *in vitro* (Fig. 2) and the levels of island excision and gene expression were quantified. Circular intermediate production measures the amount of a junction fragment of DNA that is produced when PPHGI‐1 is excised from the chromosome and forms a circular molecule (Pitman *et al*., 2005). XerC is a PPHGI‐1‐encoded integrase protein that is known to be upregulated during PPHGI‐1 excision and *topB3* is a PPHGI‐1‐encoded topoisomerase gene (described further below) (Pitman *et al*., 2005). Both ciprofloxacin and novobiocin treatments resulted in an increased expression of *avrPphB* (*p* < 0.05). However, *xerC* expression and circular intermediate production remained stable. These results suggest that the supercoiling inhibitors do not affect the level of excision of PPHGI‐1 from the bacterial chromosome but that the relaxation of supercoiling allows increased expression of island‐encoded genes, as seen for *avrPphB* and *topB3*.

**Figure 3:**
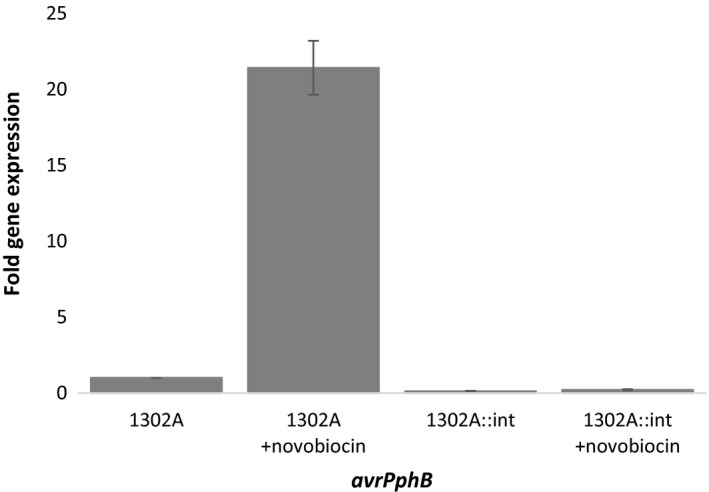
*avrPphB* expression is only affected by supercoiling inhibitors when PPHGI‐1 is excised from the chromosome. Bacterial cells were incubated in extracted bean cultivar Tendergreen apoplastic fluid for 5 h before the gene expression of *avrPphB* was measured.* avrPphB* expression is increased 21 times in wild‐type (WT) *Pph* 1302A treated with novobiocin, which can excise PPHGI‐1 from the chromosome. However, there is no difference in *avrPphB* expression between *Pph* 1302A::int (from which PPHGI‐1 is unable to excise) with and without novobiocin treatment. Results are displayed as fold expression. All data were standardised by simultaneous qPCR analysis of *acpP* expression and error bars represent standard error of the mean of three experimental replicates.

To confirm that the supercoiling inhibitors were affecting *avrPphB* expression when PPHGI‐1 is in its circular form, an insertional mutant of the *xerC* integrase gene (*Pph* 1302A::int, PPH.100) that renders PPHGI‐1 unable to excise from the chromosome (Pitman *et al*., 2005) was treated with and without 10 µg/ml novobiocin in extracted TG apoplastic fluid. The level of *avrPphB* expression increased more than 20 times as a result of treatment with novobiocin in wild‐type (WT) *Pph* 1302A, which can excise PPHGI‐1 from the chromosome (Fig. 3). However, there was no difference in *avrPphB* expression between *Pph* 1302A::int with and without novobiocin treatment, suggesting that *avrPphB* expression is only affected by supercoiling inhibitors when PPHGI‐1 is excised from the chromosome (Fig. 3). This suggests that PPHGI‐1 exists in a supercoiled form when it is excised from the chromosome causing silencing of *avrPphB*.

### The effect of topB3 disruption on genomic island PPHGI‐1 excision/loss and *avrPphB* gene expression

The gene *topB3* is a topoIII gene found in the *Pph* genomic island PPHGI‐1 that is related to *Pb*TopoIIIβ which was previously linked with regulating genomic island excision (Vanga *et al*., 2012). We hypothesised that this gene may influence the stability of the genomic island within the chromosome and thus the expression of *avrPphB*. Thus, to further characterise *topB3* for its influence on PPHGI‐1 excision, *avrPphB* expression and fitness of *Pph*, an insertional knock out of *topB3* was created (*Pph* 1302A::56).

**Figure 4:**
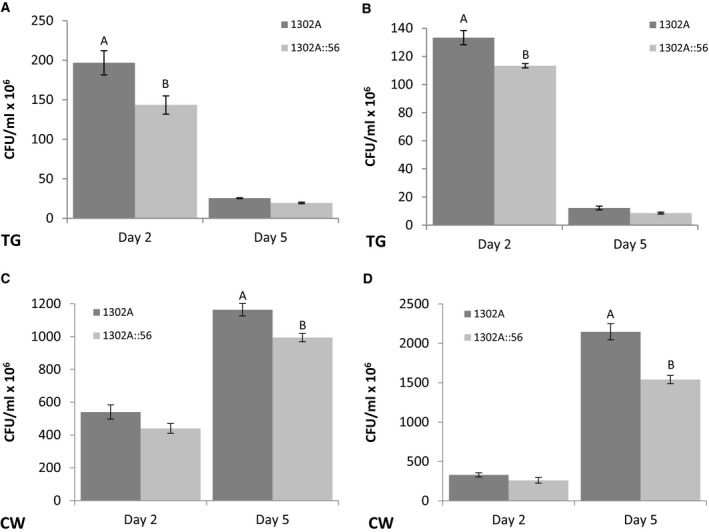
Growth of *Pseudomonas syringae* pv. *phaseolicola* 1302A::56 is lower than 1302A wild type *in planta*. A. Individual growth in resistant host Tendergreen (TG). B. Competitive growth in resistant host TG. C. Individual growth in susceptible host Canadian Wonder (CW). D. Competitive growth in susceptible host CW. Means are of three replicates ±SEM. Letters above bars indicate significant differences at p < 0.05 assessed with Student’s‐t test.

To determine if *topB3* has any pleiotropic effects on bacterial growth, growth and competition experiments were carried out. Strain 1302A::56 exhibited no significant difference in *in vitro* growth in rich medium (LB) over 24h compared to WT *Pph* 1302A (Fig. S3) when grown individually. Similarly, no difference in relative fitness was observed between *Pph* 1302AWT and *Pph* 1302A::56 in *in vitro* competition where relative fitness was equal to 0.06 (Zhang and Rainey, 2007). However, when the growth of *Pph* 1302A::56 and *Pph* 1302A WT was compared *in planta*, either as individual inoculations or in competition, a small but significant (p < 0.05) reduction in *Pph* 1302A::56 growth relative to *Pph* 1302A WT was observed after two and five days, respectively, in both the resistant cultivar TG and the susceptible cultivar Canadian Wonder (CW) (Fig. 4).

**Figure 5:**
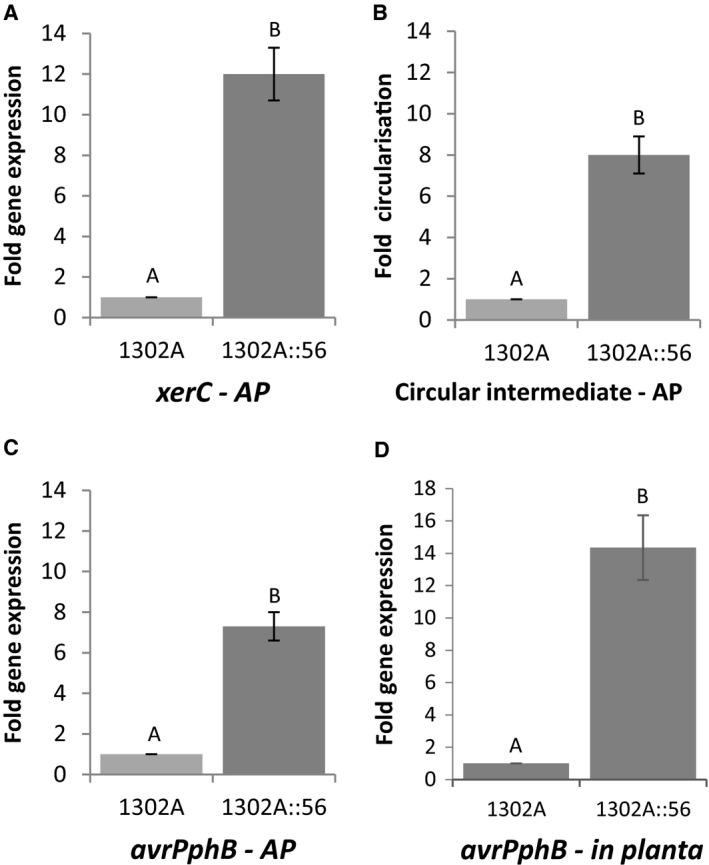
Gene expression and circular intermediate detection in a topoisomerase insertion mutant, strain *Pseudomonas syringae* pv. *phaseolicola* 1302A::56. Bacterial cells were inoculated in extracted bean cultivar Tendergreen apoplastic fluid for 5 h before gene expression of A. integrase gene *xerC* and C. effector gene *avrPphB* or B. detection of the circular intermediate was measured. Results are displayed as fold expression. D. Bacterial cells were inoculated into bean cultivar Tendergreen leaves and the plants incubated for 5 h before the apoplastic fluid was extracted and gene expression of effector gene *avrPphB* was measured. *avrPphB* and *xerC* expression were standardised by simultaneous qPCR analysis of *acpP* expression and error bars represent standard error of the mean of three experimental replicates. Letters above bars indicate significant differences at p < 0.05 assessed with Student’s t‐test. AP: extracted TG apoplastic fluid.

To investigate if *topB3* is involved in island excision, bacterial cells were incubated for five hours in an apoplast‐mimicking medium that is known to induce excision and circularisation of PPHGI‐1 (Pitman *et al*., 2005) and expression of *xerC*. qPCR was used to compare the expression level of the integrase *xerC*, which is involved in PPHGI‐1 excision, in the *topB3* mutant compared to the WT strain. The level of PPHGI‐1 circular intermediate produced was also examined. Circular intermediate production is used here as a measure of island excision from the chromosome, rather than detecting island loss using our standard bean pod assay (Pitman *et al*., 2005), because *in vitro* the island is not lost from the bacteria cell due to the absence of the selection pressure of the resistant plant. *xerC* showed an increase in the expression of the *topB3* mutant compared to the WT strain (Fig. 5A), which is over and above the level seen by the effect of the apoplast alone. This increase in *xerC* expression also correlated with an increase in circular intermediate formation (Fig. 5B). This supports the hypothesis that *topB3* is involved in the regulation of island excision via a direct or indirect effect on *xerC*. However, given the supercoiling inhibitors had no effect on either *xerC* expression or circular intermediate formation, this suggests the *topB3* mutation must be having an indirect effect on *xerC* expression. In contrast, *avrPphB*, which was upregulated in the presence of supercoiling inhibitors, also exhibited an eightfold increase in the expression of *topB3* mutant in apoplastic fluid (Fig. 5C) and this observation was confirmed *in planta* (Fig. 5D). Together, these data suggest that supercoiling appears to control the expression of *avrPphB* but not *xerC*, whereas TopB3 negatively regulates the expression of *avrPphB* and *xerC* probably independently of the effect of supercoiling.

To determine if disrupting *topB3*had any effect on *in planta* island loss, *Pph* 1302A::56 was passaged six times through leaves of bean cv. Tendergreen (TG) and 100 colonies were tested for the presence or absence of PPHGI‐1 using TG pods as a screen (Pitman *et al*., 2005). Strain *Pph* 1302A::56 showed a high level of island loss (96%), which was very similar to a control insertion mutant in a non‐coding region of PPHGI‐1 (98%) (Fig. S4). It was therefore clear that *topB3* is not essential for island loss from the cells.

## Discussion

Previous studies have shown that the genomic island PPHGI‐1 can be excised from the bacterial chromosome and that when this happens the expression of *avrPphB* is downregulated (Godfrey *et al*., 2011). We have previously speculated that the downregulation of *avrPphB* during island excision may be due to the supercoiling of the island as it becomes a separate circular molecule from the chromosome, and that this downregulation may effectively ‘hide’ the presence of this avirulence gene from the plant’s surveillance system, thereby reducing the elicitation of the HR response. In order to further examine this, we investigated the effect of supercoiling on PPHGI‐1 structure and *avrPphB* expression and also investigated an island‐encoded topoisomerase gene, *topB3*, that had been suggested in other systems to be involved in genomic island stability.

Firstly, we determined the MIC for two supercoiling inhibitors, novobiocin and ciprofloxacin, and found the concentrations of 10 and 0.5 µg/ml, respectively, to have no detectable effect on bacterial growth but to relax the supercoiling of a reporter plasmid pBBR1MCS‐2. These values compared well with other reported levels that were used to investigate supercoiling, for example several studies have shown relaxation of supercoiling with novobiocin at a level of 25 or 50 µg/ml in *Salmonella enterica* serovar Typhimurium (Ó Cróinín *et al*., 2006) and 4 and 8 µg/ml in *Helicobactor pylori* (Ye *et al*. 2007). Ciprofloxacin has been shown to relax plasmid DNA supercoiling in *Escherichia coli* K12 (Aleixandre *et al*., 1991) at a concentration of 0.5–3 µg/ml. Robillard *et al*. (2012) also demonstrated that ciprofloxacin relaxes supercoiling by 50% in *E. coli* H560, *P. aeruginosa* PA02 and *Klebsiella pneumoniae* MP100 at concentrations of 0.5, 1.0 and 0.5 µg/ml, respectively. When *Pph* 1302A was treated with novobiocin at 10 µg/ml, the expression of *gyrB* increased and this was taken as additional evidence that novobiocin affected the supercoiling as an increase in *gyrB* expression can be seen as an indicator that *gyrB* is being overexpressed to combat the effect of reduced supercoiling. A similar phenomenon was observed by Sioud *et al*. (2009) where it was found that during *Bacillus subtilis* treatment with novobiocin to inhibit gyrase‐maintained supercoiling, both *gyrA* and *gyrB* genes were upregulated.

After establishing the levels of supercoiling inhibitors that affect PPHGI‐1, we then investigated their effect on PPHGI‐1 excision from the chromosome and on *avrPphB* expression. Novobiocin and ciprofloxacin both caused an increase in *avrPphB* expression but no increase in circularisation of the island suggesting that increased expression is due to a reduction in supercoiling and not an increase in the amount of extra chromosomal island. This supports the theory that supercoiling of the excised PPHGI‐1 inhibits *avrPphB* expression. It is well established that supercoiling of DNA influences the level of transcription in cells (Pruss and Drlica, 1989; Ma and Wang 2014). For example, Ye *et al*. (2007) used genome‐wide transcript analysis under conditions of reduced supercoiling and showed both an increase and decrease in the transcription of a number of genes during relaxation of supercoiling by novobiocin. Similarly, Peter *et al*. (2004) measured the transcriptional response to loss of supercoiling due to topoisomerase gene mutation or addition of topoisomerase inhibitors in *E. coli* MG1655. They found that the transcription of 306 genes was significantly altered by a change in the level of supercoiling. More recently, Sobetzko (2016) reported that DNA supercoiling is amongst the most influential regulators of gene expression found in bacteria with more than half of all genes sensitive to supercoiling.

An insertional mutant of *topB3* (*Pph* 1302A::56), a PPHGI‐1‐encoded type IA topoisomerase gene, showed increased levels of island circularisation compared to wild type. The *Pph* 1302A::56 strain also showed increased levels of *avrPphB* expression. Since topoisomerase inhibitors were found to increase the expression of *avrPphB* when it was present in the circular intermediate, this suggests that *topB3* also contributes to the suppression of *avrPphB* expression by modulating the supercoiling of the circular intermediate. However, the unexpected observation that *avrPphB* expression was increased in the *topB3* mutant suggests a more complex regulation where TopB3 is actually having a direct effect on gene expression of *avrPphB* and not through a role in supercoiling. This would be an area for further investigation.

Vanga *et al*. (2012) showed an increase in the excision of the genomic island HA12 when they inactivated a similar island‐encoded topoisomerase gene *Pb*TopoIIIβ. These authors suggest that *Pb*TopoIIIβ may be responsible for island stability in the chromosome and this may also be the case in the current study. The role of Topo III proteins is not completely understood, but the enzyme is thought to be important for the maintenance of genomic stability (Deweese *et al*., 2008). Topo III proteins are also known to act as a potent decatenase on DNA rings that have small gaps, but are very poor at relaxing supercoils (Perez‐Cheeks *et al*., 2012).

Vanga *et al*. (2012) also observed a reduction in virulence of *Pba* with *Pb*TopoIIIβ disrupted in potato and we observed a reduction in virulence by the *topB3‐*disrupted strain on the susceptible bean cultivar CW after five days. We have previously observed that in CW PPHGI‐1 confers a fitness advantage to *Pph* 1302A (Neale *et al*., 2016) and it may be that increasing its excision and altering the gene expression of island‐encoded genes has a deleterious effect on the ability of *Pph* 1302A to cause disease. Here we also observed that the *topB3*‐disrupted strain caused an increase in the HR response in the early stages of infection as measured by a reduction in bacterial growth in the resistant cultivar (2 days).

Here we have shown that the function of a genomic island, and the expression of a gene carried by it, is affected by supercoiling inhibitors, and that supercoiling may be a way in which, once circularised, the island controls gene expression. We have also shown the importance of an island‐encoded topoisomerase gene for both island stability in the chromosome and island gene expression. However, other factors could be influencing gene expression on PPHGI‐1. Given that the integrase family of tyrosine recombinases can alter the topology of DNA (Grainge and Jayaram, 1999), then it may be possible that the *xerC* gene on the island could be affecting PPHG‐1 topology and therefore altering gene expression of island‐encoded genes. In addition to plant pathogenic bacteria, pathogens of humans and other animals contain many important GIs including SPI‐1 from *Salmonella enterica* which also encodes a T3SS (Hensel, 2004), the cag‐pathogenicity island from *Helicobactor pylori* which encodes a type IV secretion system (Wang *et al*., 2016) and the locus of enterocyte effacement genomic island in *E. coli* (Franzin and Sircili, 2015). It would therefore be of interest to see how widespread this phenomenon of regulation of island‐encoded genes being affected by the physical state of the island is and whether island‐encoded topoisomerase genes have a wider role in genomic island stability. Given the well‐established use of supercoiling inhibitors as antimicrobials in a clinical context, it will also be interesting to establish whether they could also potentially be integrated into plant disease control strategies.

## Experimental procedures

### Bacterial and plant growth conditions

*Pph* strains (Table 1) were cultured at 25^°^C for 48 h on King’s B (KB) agar plates (Difco, UK). Overnight cultures were grown in Luria–Bertani media (LB, Difco, UK) at 25^°^C. Antibiotics and supercoiling inhibitors were used at the following concentrations (μg/ml): kanamycin (Km) 50, novobiocin (1–100) and ciprofloxacin (0.5–5). Bean cultivars Tendergreen (TG) and Canadian Wonder (CW) were grown for 14 days at 23^°^C, 70% humidity with a 16 h photoperiod.

**Table 1:** Bacterial strains

Strain	Description	Source
*Pseudomonas syringae* pv. *phaseolicola* (*Pph*) 1302A	Cause of halo blight disease in bean, Race 4	Taylor *et al*. (1996)
*Pph* 1302A::56	PPHGI‐1::*Pph*TopoIIIβ	Lovell *et al*. (2009)
Pph 1302A::int	PPHGI‐1::*xerC*	Pitman *et al*. (2005)

### Plant passaging

Overnight bacterial cell suspensions were washed and the pellet resuspended in 10 mM MgCl_2_ followed by dilution to an optical density of 0.1 (OD_600_, CFU 8 × 10^7^) before being infiltrated into bean leaves via a syringe and needle. After 7 days, inoculated tissue was dissected and homogenised in 10 mM MgCl_2_. Bacteria were recovered by brief centrifugation to remove any remaining plant tissue and diluted to their original inoculated optical density, before being re‐infiltrated into fresh leaves. Recovered bacteria were plated onto KB agar at each passage and single colonies screened for their ability to cause disease symptoms or HR by inoculating them into TG bean pods using a sterile cocktail stick.

### Growth assays

For *in planta* growth assays, three replicates of each cell suspension were grown overnight in LB broth and diluted to 8 × 10^7^ CFU/ml (OD_600_ 0.1). Cells were inoculated into 14‐day‐old bean leaves via a syringe and needle either as individual cultures or 50:50 of each strain for competition assays. Plants were incubated for 48 and 120 h. Bacteria were harvested as above and total CFU/ml were counted. Fitness was calculated as per Zhang and Rainey (2007).

For *in vitro* growth assays, three replicates of each bacterial mutant were grown overnight in LB broth and diluted to 8 × 10^8^ CFU/ml (OD_600_ 1.0). Cells (100 μl) were sub‐cultured into 10 ml fresh LB broth and incubated at 25^°^C shaking (10 g). At 0, 2, 4, 6, 8, 16 and 24 h, samples were diluted and plated onto KB + Kan and total CFU/ml counted. For competition assays, 50:50 of each strain was used and bacterial cells were diluted and plated onto KB/KB + Kan every hour for 8 h and total CFU/ml counted.

### Minimum inhibitory concentrations (MICs)

MICs for novobiocin and ciprofloxacin in *Pph* were determined using doubling dilution steps from 512 to 0.0625 µg/ml and recording CFU/ml bacterial growth. 10 µg/ml novobiocin and 0.5 µg/ml ciprofloxacin were determined as concentrations that had no effect on bacterial growth.

### Separation of plasmid topoisomers by chloroquine gel electrophoresis

The reporter plasmid pBBR1MCS‐2 (Kovach *et al*., 1994) was electroporated into *Pph* 1302A following the method of Keen *et al*. (1992). This strain was then treated with 1, 5, 10, 50 µg/ml novobiocin or 0.1, 0.25, 0.5, 1 µg/ml ciprofloxacin for 4 h. Plasmid DNA was then extracted using the Qiagen mini‐prep plasmid kit and visualised on a 1% agarose gel containing 2.5 µg/ml chloroquine as per Higgins *et al*. (1988).

### PCR and qPCR

For PCR, a standard 25 μl PCR mix was used that consisted of the following: 1 μl bacterial culture, 0.5 μM oligonucleotide primers (Table 2), 12.5 μl mastermix (Taq PCR mastermix, Qiagen) and 9.5 μl sterile deionised water. Standard PCR cycling conditions consisted of 94^°^C for 10 min, followed by 30 cycles of 94^°^C for 30 s, X^°^C for 30  s (dependent on the annealing temperature of primers) and 72^°^C for 1 min, followed by a final extension step of 72^°^C for 10 min.

**Table 2:** Oligonucleotide primers

Primer name	Description	Sequence 5′–3′
acpP	Housekeeping gene	Forward TTGGCGTCAAATCAGAAGAG
		Reverse GCTTCTTCGTCAGGGATTTC
		Probe ACCTGGGCGCTGACTCCCTG[Link]
gyrB	*gyrB* gene	Forward GATGATGGAATCGGTGTCGAA
		Reverse TTGGTGAAGCACAACAGGTTCT
		Probe CCCTGCAGTGGAACGACAGCTTCA[Link]
xerC	PPHGI‐1 encoded *xerC* intergrase	Forward CGACGATACGGCCTCCAA
		Reverse AAAGGTGCGGTCGACATCA
		Probe CCCCCTATAGCGGAGCGTCTGGAA[Link]
QavrPphB	PPHGI‐1 encoded effector gene	Forward CCCATTCCTGGCAATGACA
		Reverse TTACGCCTGAAGAGGATGCA
		Probe TGGGCGATAAAGGG[Link]
QCI	Circular intermediate	Forward CATGGGCCTTCCAGATTTTC
		Reverse CTGCGGTTTGGGATACTGAAC
		Probe CGTAACGCTGAGGCAGGCCCC[Link]
avrPphB	PPHGI‐1 encoded effector *avrPphB*	Forward GCGATTGCGTGTCCTTGA
		Reverse CTGTAAGACCTGAGCCTG

^a^Probes labelled with 5′ FAM and 3' TAMRA TaqMan dyes.

After amplification, a 20 μl sample was visualised on a 0.7% agarose gel with 2 μg/ml ethidium bromide in comparison to a DNA molecular marker (Hyperladder, Bioline).

qPCR was used to quantify the expression levels of *gyrB, avrPphB, xerC* and the amount of circular intermediate produced *in vitro.* Bacterial cells were incubated in 50:50 1 × M9 Minimal medium with 20% glucose (Sambrook and Russell, 2001) and TG apoplastic fluid. Apoplastic fluid was extracted using the method described by Lovell *et al*. (2009) where appropriate cultures were spiked with 1–10 µg/ml novobiocin or 0.5–5 µg/ml ciprofloxacin. After 5 h shaking at 25^°^C, cells were harvested using RNA protect reagent (Qiagen, UK) for RNA extraction or DNA lysis solution (Gentra Systems, UK) for DNA extraction. For quantification of *gyrB, avrPphB* and *xerC* expression, RNA was extracted using the RNAeasy kit (Qiagen) followed by a second DNase step of 15 min at 37^°^C (Promega, UK) (Godfrey *et al*., 2011). cDNA was synthesised using the TaqMan reverse transcription kit (Qiagen). For circular intermediate quantification, DNA was extracted using the Puregene total DNA extraction kit (Gentra Systems) (Godfrey *et al*., 2011). For *in planta* expression, whole TG leaves were inoculated with 0.1 OD_600_ *Pph* 1302A and incubated for 5 h before apoplastic fluid was extracted. X‐fold change in gene expression was calculated using WT as the calibrator and *acpP* as the internal control (Table 2). The gene *acpP* has been demonstrated to be a stably expressed internal reference gene in *Pseudomonas* (Lenz *et al*., 2008; Qi *et al*., 2014). For the current study, it was tested under a variety of conditions and showed no change in expression (Fig. S5).

## Author contributions

The conception or design of the study: HN, DLA.

The acquisition, analysis, or interpretation of the data: HN, DLA, RWJ, GP.

Writing of the manuscript HN, DLA, RWJ, GP.
