# Supplementary figures and images for: Supercoiling of an excised genomic island represses effector gene expression to prevent activation of host resistance
Source: Mol Microbiol. 2018 Oct 3;110(3):444–54. doi: 10.1111/mmi.14111 (PMC6220960; doi:10.1111/mmi.14111)

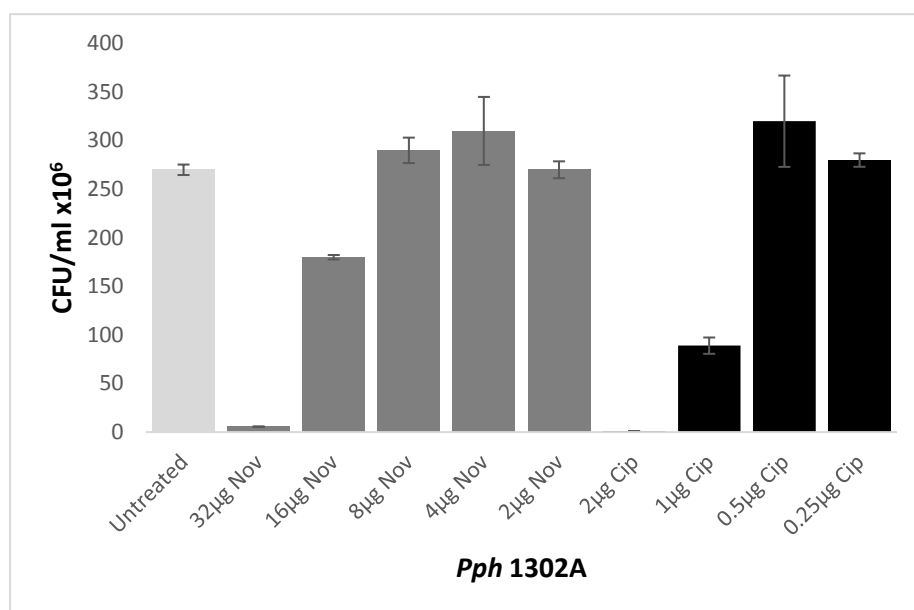

**Fig. S1.**

Supplement: Supplementary file 1 [file MMI-110-444-s001.pdf]

**Figure S2**

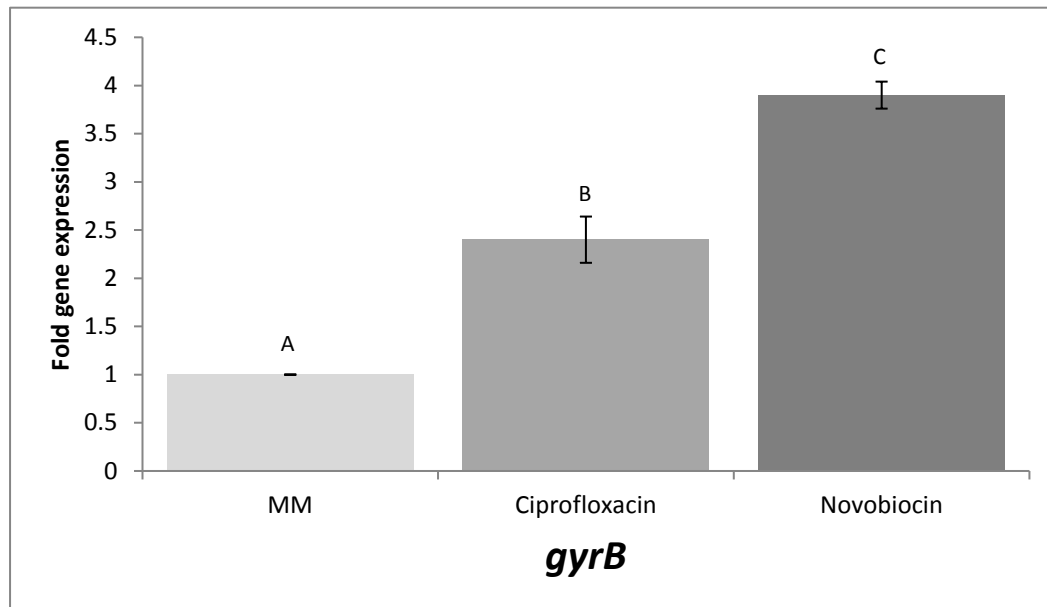

Supplement: Supplementary file 2 [file MMI-110-444-s002.pdf]

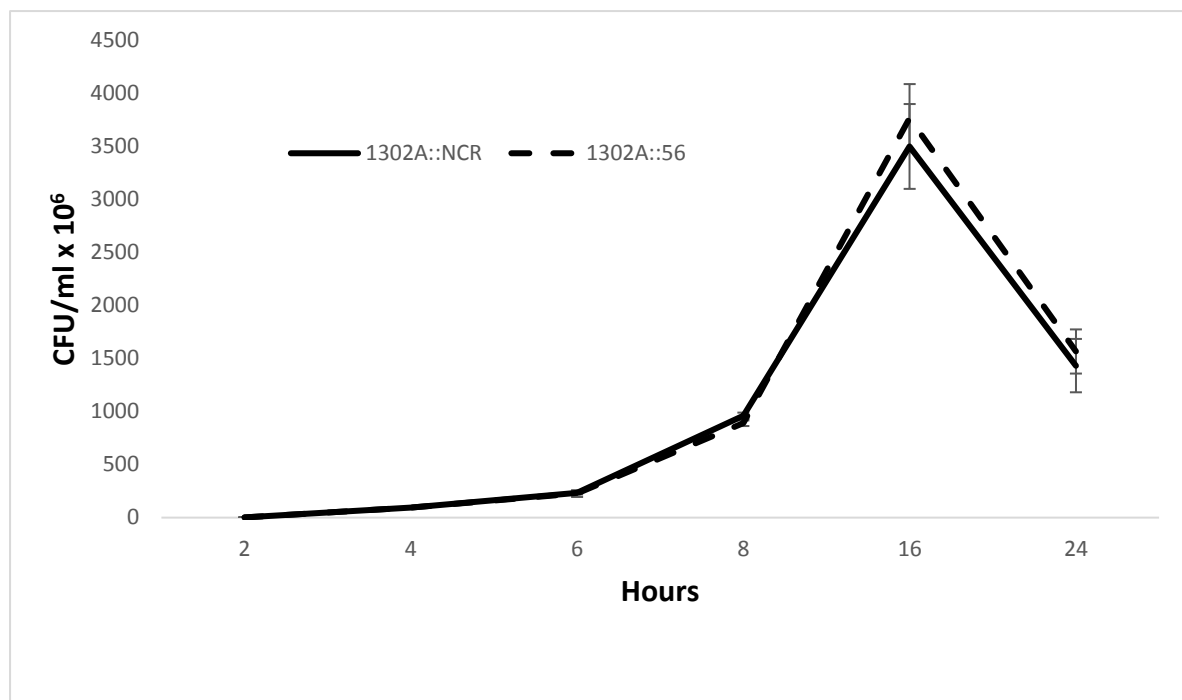

**Figure S3**

Supplement: Supplementary file 3 [file MMI-110-444-s003.pdf]

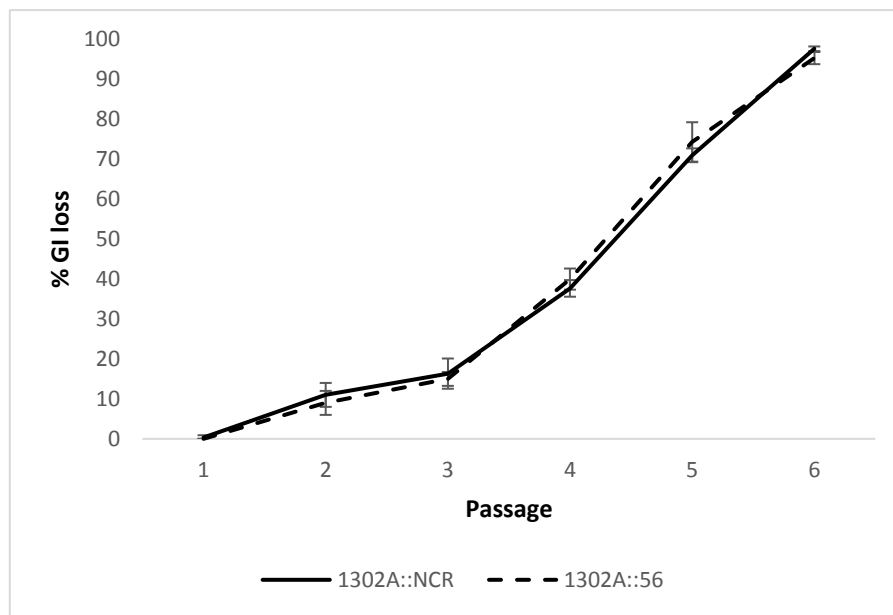

**Figure S4**

Supplement: Supplementary file 4 [file MMI-110-444-s004.pdf]

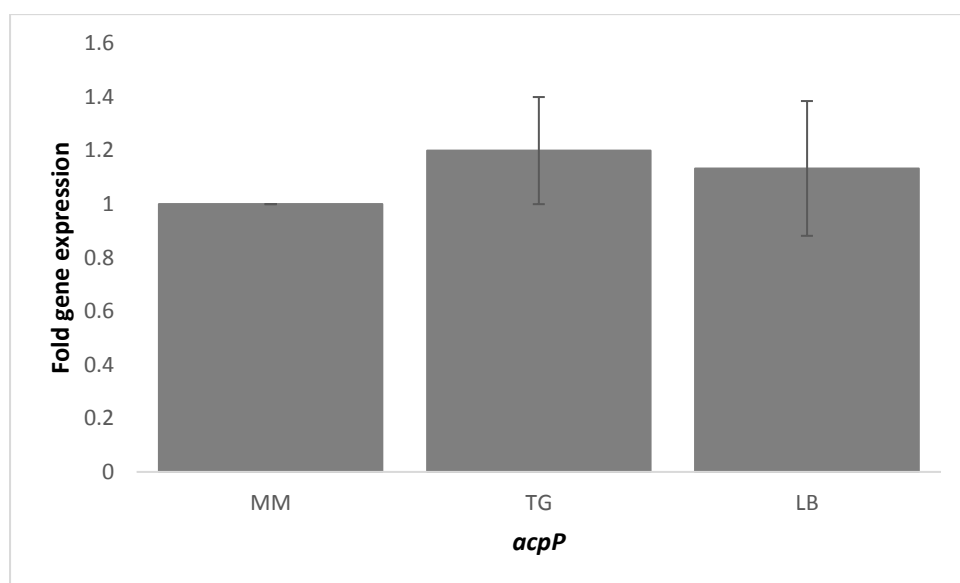

**Figure S5**

Supplement: Supplementary file 5 [file MMI-110-444-s005.pdf]
